# Causal association of circulating immune cells and lymphoma: A Mendelian randomization study

**DOI:** 10.1515/med-2024-0984

**Published:** 2024-07-15

**Authors:** Feixiang Wang, Guoxin Huang, Yuqing Luo, Kaixin Xiong, Ying Liu, Yao Wang

**Affiliations:** Medical Oncology Department, Affiliated Cancer Hospital & Institute of Guangzhou Medical University, Guangdong, Guangzhou, 510095, China; Department of Evidence-Based Medicine Center, Xiangyang No.1 People’s Hospital, Hubei University of Medicine, Xiangyang, Hubei, 441000, China; Department of Evidence-Based Medicine Center, Xiangyang No.1 People’s Hospital, Hubei University of Medicine, No. 15, Jiefang Road, Xiangyang, Hubei, 441000, China; Medical Oncology Department, Affiliated Cancer Hospital & Institute of Guangzhou Medical University, No. 78, Hengzhigang, Yuexiu District, Guangdong, Guangzhou, 510095, China

**Keywords:** circulating immune cells, malignant lymphoma, mendelian randomized study, pathogenic factors, protective factors

## Abstract

**Background:**

Malignant lymphoma (ML) is a group of malignant tumors originating from the lymphatic hematopoietic system. Previous studies have found a correlation between circulating immune cells and ML. Nonetheless, the precise influence of circulating immune cells on ML remains uncertain.

**Methods:**

Based on publicly available genetic data, we explored causal associations between 731 immune cell signatures and ML risk. A total of four types of immune signatures, median fluorescence intensities, relative cell, absolute cell, and morphological parameters were included. Primary analysis was performed using inverse variance weighting (IVW) to assess the causal relationship between circulating immune cells and the risk of ML. Sensitivity analysis was conducted using Cochran’s *Q* test, the Mendelian randomization Egger regression intercept test, and leave-one-out analysis.

**Results:**

ML had a statistically significant effect on immunophenotypes. Twenty-three immunophenotypes were identified to be significantly associated with Hodgkin lymphoma risk through the IVW approach, and the odds ratio values of CD64 on CD14^−^ CD16^+^ monocyte [2.31, 95% confidence interval (CI) = 1.41–3.79, *P*1 = 0.001], IgD^+^ CD24^+^ B-cell %lymphocyte (2.06, 95% CI = 1.13–3.79, *P*1 = 0.018), B-cell %lymphocyte (1.94, 95% CI = 1.08–3.50, *P*1 = 0.027), CD24^+^ CD27^+^ B-cell %lymphocyte (1.68, 95% CI = 1.03–2.74, *P*1 = 0.039), and CD14^+^ CD16^−^ monocyte %monocyte (1.60, 95% CI = 1.15–2.24, *P*1 = 0.006) ranked in the top five. Eleven immunophenotypes were identified to be significantly associated with non-Hodgkin lymphoma risk, CD86 on granulocyte (2.35, 95% CI = 1.18–4.69, *P*1 = 0.015), CD28^−^CD8^+^ T-cell absolute count (1.76, 95% CI = 1.03–2.99, *P*1 = 0.036), CCR2 on myeloid dendritic cell (CD24^+^ CD27^+^ B cell, 95% CI = 1.02–1.66, *P*1 = 0.034), CD3 on effector memory CD8^+^ T cell (1.29, 95% CI = 1.02–1.64, *P*1 = 0.012), and natural killer T %lymphocyte (1.28, 95% CI = 1.01–1.62, *P*1 = 0.046) were ranked in the top five.

**Conclusion:**

This study presents compelling evidence indicating the correlation between circulating immune cells and lymphoma, thus providing guidance for future clinical research.

## 1 Introduction

Malignant lymphoma (ML) is a group of malignancies originated from the lymphatic hematopoietic system. Lymphomas are classified into two categories based on pathology, clinical characteristics, and prognosis: non-Hodgkin lymphoma (NHL) and Hodgkin lymphoma (HL) [1]. According to GLOBOCAN 2020 data, there were 83,087 new cases of HL and 544,352 new cases of NHL worldwide, accounting for the 13th most common type of new cancer cases. In 2020, there were 259,793 deaths due to NHL globally, ranking 12th in cancer mortality [2]. There are over 100 subtypes of ML, characterized by high heterogeneity. Most MLs are treated with targeted therapy and chemotherapy, sometimes combined with radiotherapy. High-risk or relapsed/refractory ML patients may also need hematopoietic stem cell transplantation to further consolidate the therapeutic effect [3]. In recent years, with the rapid development of immunotherapy, treatment methods such as monoclonal antibodies, antibody–drug conjugates, immune checkpoint inhibitors, and chimeric antigen receptor T-cell immunotherapy have gradually been integrated into the first-line regimens and treatment options for relapsed/refractory ML patients [1].

The incidence and progression of ML are closely related to the immune system. Previous studies have shown that in ML, the loss of major histocompatibility complex (MHC) molecules is common, leading to decreased antigen presentation and impaired appropriate immune response [4]. Additionally, ML often overexpress programmed death-ligand 1 (PD-L1; CD274) and PD-L2 (CD273), thereby protecting themselves from the effects of activated T cells, with expression usually driven by viral or genetic factors [5,6]. Although multiple immune cell populations exist in the tumor microenvironment (TME) of ML, many of these cell types do not target malignant B cells but have suppressive and regulatory effects on the immune response instead. Moreover, ML cells secrete a variety of cytokines that directly inhibit T-cell function or induce T-cell exhaustion. Studies have found that increased levels of IL-10 can induce the expansion of myeloid-derived suppressor cells and inhibit T-cell proliferation [7,8].

Increasing evidence suggests that immune cell populations in the TME of ML have an impact on the incidence, progression, and treatment response of the disease [9]. CD8^+^ T cells express a receptor named natural killer group 2 member D (NKG2D), whose ligands are MHC complex class I polypeptide-related sequence (MIC). MIC-A and MIC-B were widely expressed ligands in human solid tumors [10]. Studies have found that tumor cells can evade NKG2D-mediated immune responses by releasing soluble MIC [11]. NK cells play an important role in recruiting dendritic cells to tumors, thereby enhancing the induction of CD8^+^ T-cell responses. The activation of NKG2D receptors and their MIC-A and MIC-B ligands on tumor cells offered opportunities for therapeutic intervention [12]. Natural killer T cells (NKT) are a unique subgroup of T cells that possess both T-cell receptors (TCR) and NK cell receptors, acting as a bridge between innate and adaptive immunities. NKT cells produce large amounts of cytokines and exhibit cytotoxic effects similar to NK cells [13].

Mendelian randomization (MR) is an epidemiological method of causal inference analysis based on the law of Mendelian independent assortment. As genetic variations are randomly allocated at conception before the onset of disease, MR is an effective tool for identifying causal relationships independent of confounding factors and avoiding reverse causality [14]. Previous observational studies have found many associations between immune cell characteristics and ML, supporting the hypothesis of a correlation between the two [15]. In this study, we conducted a comprehensive two-sample MR analysis to determine the causal relationship between immune cell characteristics and ML.

## 2 Materials and methods

### 2.1 Study design

**Figure 1:**
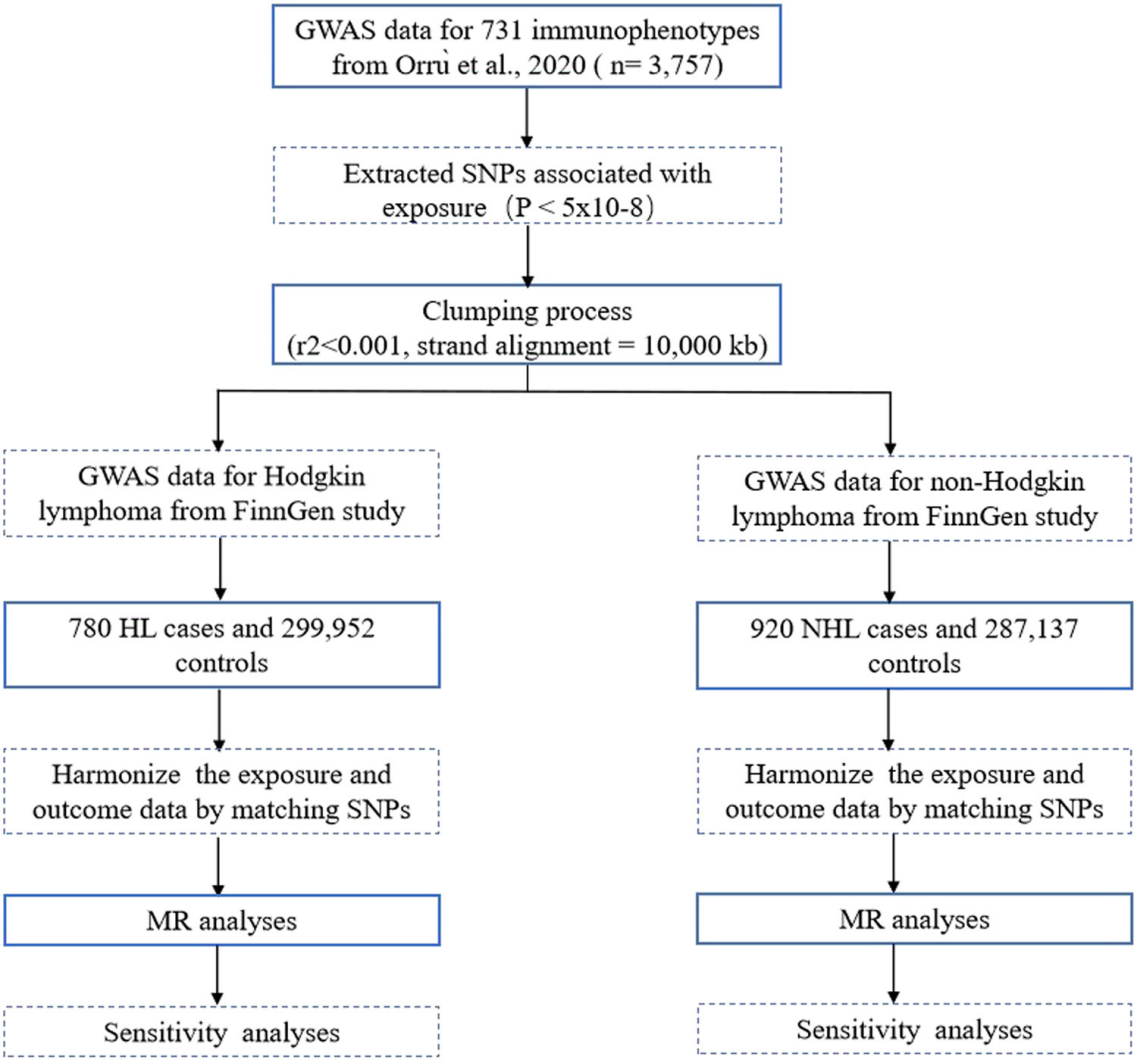
Study design of MR study between 731 immunophenotypes and lymphoma. MR analyses: IVW, Mendelian randomization Egger regression (MR-Egger), weighted median, and weighted mode. Sensitivity analyses: Cochran’s *Q* test, the MR-Egger intercept test, and LOO analysis.

In this study, we applied two-sample MR analyses to explore the causal relationship between circulating immune cells and the risk of HL and NHL (Figure 1). Valid causal inferences in the MR study adhere to three core assumptions: (1) genetic variations are strongly related to the exposure; (2) genetic variations are not directly related to the outcome; and (3) genetic variations are not related to any confounding factors outside of the exposure-outcome pathway [16].

### 2.2 Data sources for immune cell signatures

Genetic variations data correlated with immune cell signatures as exposure variables were derived from the updated available meta-analysis of genome-wide association studies of immunophenotypes from Orrù et al. [17]. Original genome-wide association study (GWAS) data of immune cell signatures were derived from 3,757 individuals within the Sardinian founder population analyzed by flow cytometry [18,19]. The study detected immune cells with 731 immunophenotypes, including T cells, B cells, natural killer cells, regulatory T cells, dendritic cells, monocytes, and myeloid cells by utilizing TBNK panels, Treg panels, maturation stages of T-cell panels, DC panels, B-cell panels, monocyte panels, and myeloid cell panels. Four parameters were counted for cellular phenotyping [20], including median fluorescence intensities (*n* = 389), relative cell (*n* = 192), absolute cell (*n* = 118), and morphological parameters (*n* = 32).

### 2.3 Data sources for HL and NHL

GWAS data with HL and NHL were derived from the FinnGen study. Launched in Finland in 2017, the FinnGen study relied on Finnish digital health record data provided by national health registries and large-scale genomic data from the Finnish biobanks [21]. The outcome GWAS data for HL consisted of 780 HL cases and 299,952 controls of European ancestry (https://r9.risteys.finngen.fi/endpoints/CD2_HODGKIN_LYMPHOMA_EXALLC), and the NHL GWAS data included 920 NHL cases and 287,137 controls of European ancestry (https://r9.risteys.finngen.fi/endpoints/C3_NONHODGKIN_EXALLC).

### 2.4 Selection of instrumental variables

We performed a series of quality controls to select appropriate genetic variable instruments. We commonly use single-nucleotide polymorphisms (SNPs) to represent genetic variations, which refer to aggregation of the diversity of DNA sequences at the genome level caused by variation in a single nucleotide. First, we selected SNPs related to immune cell signatures with a genome-wide significance level threshold of 5 × 10^−8^. In addition, we removed the SNPs (*r* ^2^ < 0.001 and strand alignment = 10,000 kb) with linkage disequilibrium based on 1,000 genomes project to obtain mutually independent SNPs [22]. Besides, to evaluate whether there was a weak instrumental variables bias, we utilized the formula to calculate the *F* statistic: *F* = [(*N* − *k* − 1)]/*k* × [*R*2/(1 − *R*2)], where *R*2, *N*, and *k* stand for the proportion of the variability of the exposure explained by each SNP, the sample size of the exposure GWAS, and the number of SNPs selected [23]. When the overall *F* statistic is above 10, it can indicate that the selected instrumental variable is appropriate.

### 2.5 Statistical analysis

Genetic variations extracted from immune cells need to be matched in GWAS data for HL and NHL. We adopted four methods including inverse variance weighted (IVW), MR Egger, weighted median, and weighted mode. IVW is the major method and the others are used as supplementary methods [16]. Burgess et al. reported that in MR analyses, traditional IVW was regarded as the most efficient method [24]. IVW results would be unbiased if horizontal pleiotropy had not existed. Based on the assumption of Instrument Strength Independent of Direct Effect (InSIDE), Mendelian randomization Egger (MR-Egger) used intercept terms to evaluate the existence of horizontal pleiotropy [25]. The weighted median provided accurate estimates of causality under the hypothesis of valid SNPs (≥50%). If the InSIDE hypothesis is violated, the weighted model has higher efficacy and less bias than MR-Egger. These methods were employed to determine MR estimates for each risk factor when three assumptions of MR were fulfilled [26]. Odds ratios (ORs) were described by the increase in risk factor levels per standard deviation. With SNPs ≥3, IVW used the random-effects meta-analysis to derive overall MR estimates by synthesizing the Wald estimate for each SNP. With SNPs ＜3, only IVW was used and the fixed-effects model was selected [27]. When we calculated estimates of the causal effect of each immunophenotypes on lymphoma, the significance level of each individual test was expressed using *P*1, with a significance threshold of 0.05. *P*1 and *P*1 denoted the *P*-value for heterogeneity and pleiotropy respectively. In this study, 731 statistical tests were performed simultaneously, and it was important to correct the *P*-value for each immune cell signature by the multiple test correction to make the overall error rate less than or equal to the corrected significance threshold. We employed the Benjamini–Hochberg (FDR) method to obtain the corrected *P*-values expressed as *P*1, with a significant threshold of 0.05/731 (6.84 × 10^−5^).

### 2.6 Sensitivity analyses

We performed IVW and MR-Egger to detect the presence of heterogeneity. Cochran’s *Q* test was adopted for IVW for the assessment of heterogeneity, and *P*-value >0.05 indicates no heterogeneity. The presence of the horizontal pleiotropy was determined by MR-Egger regression intercept test with *P*-value ≤0.05 [28]. The pleiotropy test was not performed with SNPs <3. The fixed effects model or random effects model was selected according to heterogeneity. Leave-one-out (LOO) was utilized to identify pleiotropic outlier SNPs that significantly affect causal effects [29]. A series of sensitivity analyses without contradictory results ensured the robustness of the results by examining the underlying MR assumptions. All statistical calculations were carried out on the R software (version 4.3.0 of this installation). MR analyses were achieved through R software package Two Sample MR. The forest plots were generated using forest plot package (version 2.0.0).

## 3 Results

### 3.1 Causal effects of immune cell signatures on HL

**Figure 2:**
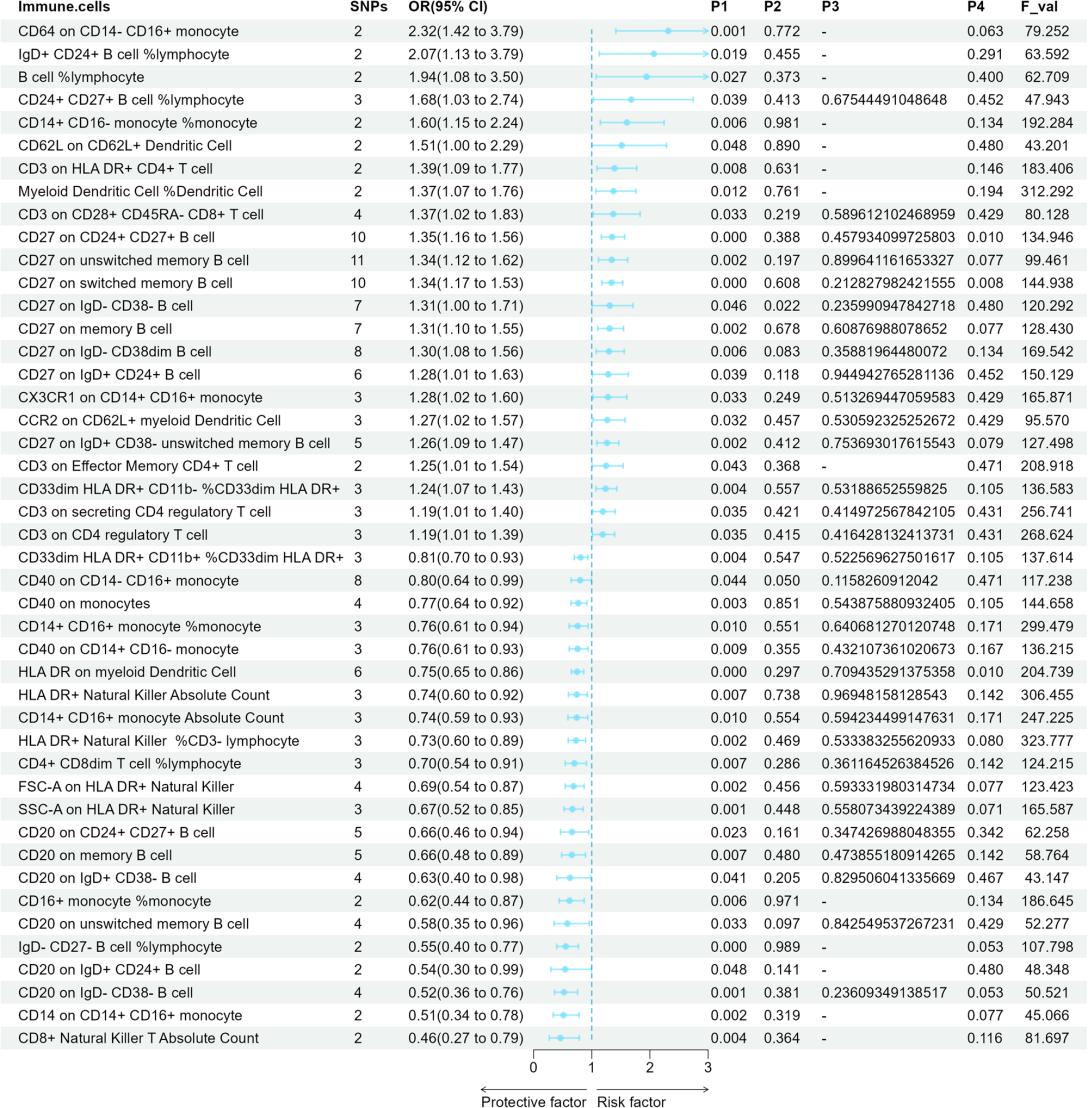
Forest plot for causal effects of circulating immune cells on the risk of HL by IVW. OR: odds ratio; CI: confidence interval; HL: Hodgkin lymphoma; *P*1: *P*-values for MR causal effects; *P*1: *P*-values for heterogeneity; *P*1: *P*-values for pleiotropy; *P*1: *P*-values corrected for the FDP method.

The causal effect of immune cell signatures on HL was estimated by the forementioned four methods: IVW, MR-Egger, weighted median, and weighted mode. Immune cell signatures with the significance level of *P*-values via IVW are summarized in Figure 2. Higher OR value (>1) represents a higher risk of pathogenicity of this immune cell signature for lymphoma. Conversely, a lower OR value (0–1) means that this immune cell signature was more protective against lymphoma. Clinical prevention and treatment of lymphoma can be guided by identifying immune cell signatures with higher OR values. We found that increases in 23 immunophenotypes were associated with higher risks of HL through the IVW approach. Among them, the OR values of CD64 on CD14^−^ CD16^+^ monocyte (2.31, 95% CI = 1.41–3.79, *P*1 = 0.001), IgD^+^ CD24^+^ B-cell %lymphocyte (2.06, 95% CI = 1.13–3.79, *P*1 = 0.018), B-cell %lymphocyte (1.94, 95% CI = 1.08–3.50, *P*1 = 0.027), CD24^+^ CD27^+^ B-cell TD CD4^+^ T-cell %lymphocyte (1.68, 95% CI = 1.03–2.74, *P*1 = 0.039), and CD14^+^ CD16^−^ monocyte %monocyte (1.60, 95% CI = 1.15–2.24, *P*1 = 0.006) ranked in the first five places. It has CD64 on CD14^−^ CD16^+^ monocyte individuals will have a 2.31 times greater risk of HL than individuals without this immunophenotype. Similarly, individuals with IgD^+^ CD24^+^ B-cell %lymphocyte, B-cell %lymphocyte, CD24^++^ CD27^+^ B-cell TD CD4^+^ T-cell %lymphocyte, and CD14^+^ CD16^−^ monocyte %monocyte will have a 2.06×, 1.94×, 1.68×, and 1.60× greater risk of developing HL compared to those without those immunophenotypes, respectively. In addition, the increases in 22 immunophenotypes in total were associated with negative effects on the risks of HL via the IVW. Among them, the OR values of CD8^+^ NKT Absolute Count (0.46, 95% CI = 0.27–0.79, *P*1 = 0.004), CD14 on CD14^+^ CD16^+^ monocyte (0.51, 95% CI = 0.33–0.78, *P*1 = 0.002), CD20 on IgD^−^CD38^−^ B-cell (0.52, 95% CI = 0.36–0.77, *P*1 = 0.001), CD20 on IgD^+^ CD24^+^ B-cell (0.54, 95% CI = 0.30–0.99, *P*1 = 0.048), and IgD^−^ CD27^−^ B-cell %lymphocyte (0.55, 95% CI = 0.40–0.77, *P*1 = 0.000) ranked in the last five places. It has CD8^+^ NKT T Absolute Count, CD14 on CD14^+^ CD16^+^ monocyte, CD20 on IgD^−^CD38^−^ B-cell, CD20 on IgD^+^ CD24^+^ B-cell, and IgD^−^ CD27^−^ B-cell %lymphocyte individuals will have 0.46 times, 0.51 times, 0.52 times, 0.54 times, and 0.55 times lower risk of HL than individuals without these immunophenotypes, respectively. It is noteworthy that similar significant associations with the other three methods (MR-Egger, weighted median, weighted mode) were observed between CD27 on CD24^+^ CD27^+^ B cell, CD27 on switched memory B cell, CD40 on CD14^−^ CD16^+^ monocyte, and HL (Table S1).

When CD27 on IgD^−^ CD38^−^ B cell acted as the exposure, the multiplicative random-effects model of the IVW was accepted with the presence of heterogeneity. When adopting the Benjamini–Hochberg method to adjust for multiple comparisons across immune cell categories, we did not discover any results that reached the FDR-corrected significance level (*P*1 < 6.84 × 10^−5^). Notably, there was suggestive evidence (*P*1, 6.84 × 10^−5^ − 0.05) of causal correlations between CD27 on CD24^+^ CD27^+^ B cell (*P*1 = 0.010), CD27 on switched memory B cell (*P*1 = 0.008), HLA DR on myeloid DC (*P*1 = 0.010), and HL. No other significant heterogeneity was apparent through sensitivity analyses (Figure 2); the MR-Egger intercept test indicated that there was no evidence of the presence of horizontal pleiotropy (Figure 2).

### 3.2 Causal effects of immune cell signatures on NHL

**Figure 3:**
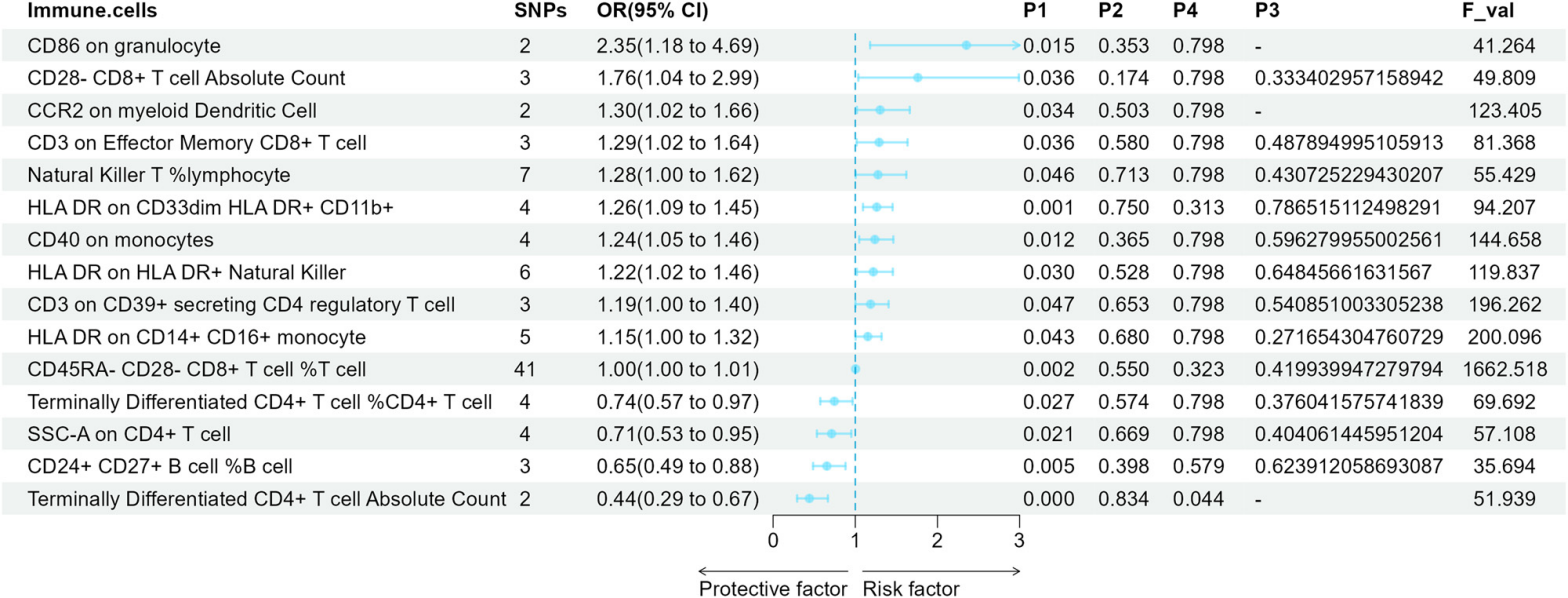
Forest plot for causal effects of circulating immune cells on the risk of NHL by IVW. OR: odds ratio; CI: confidence interval; NHL: non-Hodgkin lymphoma; *P*1: *P*-values for MR causal effects; *P*1: *P*-values for heterogeneity; *P*1: *P*-values for pleiotropy; *P*1: *P*-values corrected for the FDP method.

The causal effects of immune cell signatures on NHL were also estimated by the four methods, and Figure 3 summarizes the immunophenotypes for which results were significant through the IVW approach. A total of 11 immunophenotypes were observed to increase the risk of developing NHL via IVW. The OR values were ranked in the top five as CD86 on granulocyte (2.35, 95% CI = 1.18–4.69, *P*1 = 0.015), CD28-CD8^+^ T-cell Absolute Count (1.76, 95% CI = 1.03–2.99, *P*1 = 0.036), CCR2 on myeloid dendritic cell (1.30, 95% CI = 1.02–1.66, *P*1 = 0.034), CD3 on effector memory CD8^+^ T cell (1.29, 95% CI = 1.02–1.64, *P*1 = 0.012), and NKT %lymphocyte (1.28, 95% CI = 1.01–1.62, *P*1 = 0.046). Individuals with CD86 on granulocyte, CD28-CD8^+^ T-cell Absolute Count, CCR2 on myeloid dendritic cell, CD3 on effector memory CD8^+^ T cell, and NKT %lymphocyte will have a 2.35×, 1.76×, 1.30×, 1.29×, and 1.28× increased risk of developing NHL compared to those without those immunophenotypes, respectively. The other three methods derived similar positive trends, but not all results were significant (Table S2). In addition, the increases of CD24^+^ CD27^+^ B-cell %B cell (OR = 0.65, 95% CI = 0.49–0.88, *P*1 = 0.005), SSC-A on CD4^+^ T cell (OR = 0.71, 95% CI = 0.53–0.95, *P*1 = 0.021), terminally differentiated CD4^+^ T-cell %CD4^+^ T cell (OR = 0.75, 95% CI = 0.57–0.97, *P*1 = 0.027), and terminally differentiated CD4^+^ T-cell Absolute Count (OR = 0.75, 95% CI = 0.57–0.97, *P*1 = 0.027) were associated with negative effects on NHL via the IVW. Individuals with these five immunophenotypes will have 0.46, 0.51, 0.52, 0.54, and 0.55 times the risk of NHL compared to those without, respectively. Similar negative meaningful associations with the weighted median method were observed between CD24^+^ CD27^+^ B-cell %B cell, SSC-A on CD4^+^ T cell, TD CD4^+^ T-cell %CD4^+^ T cell, and NHL (Table S2). We observed that, unlike the negative association for HL, the increase in CD40 on monocytes was associated with a lower risk of NHL.

Since the number of SNPs was two when CCR2 on myeloid DC, CD86 on granulocyte, and TD CD4^+^ T-cell AC acted as the exposure, we used only the fixed-effects model of the IVW. When adopting the Benjamini–Hochberg method to adjust for multiple comparisons across immune cell categories, we did not discover any results that reached the FDR-corrected significance level. Remarkably, it provided evidence that TD CD4^+^ T-cell AC was considered to be causally associated with NHL (*P*1 = 0.044, 6.84 × 10^−5^ − 0.05). By sensitivity analysis, no significant heterogeneity was identified (Figure 3). No indication of potential horizontal pleiotropy being disrupted was discovered (Figure 3).

## 4 Discussion

This comprehensive two-sample MR analysis has provided novel insights into the causal relationships between circulating immune cell signatures and the risk of HL and NHL. Our findings elucidate the complex immunological landscape that underpins lymphoma pathogenesis, reinforcing the pivotal role of immune cells in cancer biology. Importantly, the identification of 23 and 11 immunophenotypes significantly associated with HL and NML risk, respectively, underscores the specificity of immune responses in different lymphoma subtypes.

Our study found that in HL, major pathogenic factors include CD64 on CD14^−^ CD16^+^ monocytes and the percentage of CD14^+^ CD16^−^ monocytes in monocytes. Circulating monocytes can be divided into three subgroups: (1) approximately 90% are CD14^++^ CD16^−^, which is known as classical monocytes, (2) CD14^++^ CD16^+^, comprising intermediate monocytes with high CD14 and low CD16 expression, and (3) CD14^−^/lowCD16^+^ are non-classical monocytes, characterized by relatively lower CD14 expression and higher CD16 expression [30]. Wong et al. reported that classical monocytes produce the highest levels of IL-6, IL-10, CCL2, and granulocyte colony-stimulating factor. The non-classical subgroup secretes the highest levels of inflammatory cytokines like TNF-α and IL-1β. Additionally, intermediate monocytes produce these cytokines and chemokines at moderate or lowest levels [31]. Previous studies have found that levels of intermediate and non-classical monocytes are significantly increased in patients with acute leukemia, which is associated with progression [32]. Furthermore, in patients with chronic myelomonocytic leukemia (CMML), there is an increased proportion of classical monocytes in peripheral blood. Classical monocyte levels are lower in CMML patients which is sensitive to hypomethylating agents [33]. These findings are consistent with our results, indicating that monocytes are unfavorable influencing factors in tumor pathogenesis.

In HL, the primary protective factors include the CD8^+^ NKT absolute count and the percentage of IgD^−^ CD27^−^ B cells in lymphocytes. Based on differential expression of IgD and CD27, human B cells can be divided into four subgroups. IgD and CD27 double negative B (DN-B) cells constitute a heterogeneous group of B cells, which is initially described in association with aging and systemic lupus erythematosus [34]. Recently, studies have found an increase in DN-B-cell frequency in various chronic diseases, including infectious and autoimmune diseases, neurological disorders, obesity, and cancer. DN-B cells are present not only in the bloodstream but also at sites of inflammation [35,36,37]. However, their specific role as protective factors in HL remains to be further explored.

In NHL, the primary pathogenic factors include CCR2 on myeloid dendritic cells and the percentage of NKT lymphocytes. Previous research has found that CCR2 can exert immunosuppressive effects by interfering with the maturation of dendritic cells in breast cancer. Tumors lacking CCR2 expression have more mature dendritic cells and infiltrating activated CD8^+^ cytotoxic T cells [38]. Overexpression of CCR2 has been identified as a poor prognostic predictor in diffuse large B-cell lymphoma (DLBCL). It plays a significant role in the development of DLBCL by promoting cell proliferation, migration, and anti-apoptotic activities [39], although its mechanisms in the immune system remain unclear. NKT cells are divided into three subtypes: type I classical NKT cells with a constant TCRα receptor can differentiate into NKT1, NKT2, and NKT17 subgroups. Type II non-classical NKT cells lack a constant TCR were often found in the human bone marrow and liver. These cells have immunosuppressive effects and may increase tumor progression and metastasis [40]. We speculated that type II NKT cells might be a major pathogenic factor in NHL.

The primary protective factors in NHL include the percentage and absolute count of terminally differentiated CD4^+^ T cells in CD4^+^ T cells. Terminally differentiated CD4^+^ T cells may represent an aspect of immune-senescence, having reached the final stage of cell differentiation while retaining certain immune capabilities, such as secreting cytokine IFN-γ and differentiating into CD4^−^ CTLs to combat viruses [41]. This immune capability can not only combat disease progression but also cause tissue inflammation, such as maintaining the infectivity of HTLV-1 or promoting inflammatory aging of the myocardium, thereby advancing disease progression [42,43].

The use of MR analysis strengthens the causal inference drawn from our results, mitigating confounding factors often encountered in observational studies. However, it is crucial to acknowledge the limitations inherent in this approach. First, the assumption of no pleiotropy may not hold for all genetic variants used as instruments, potentially biasing the estimates. Second, our analysis is contingent upon the accuracy and completeness of the immunophenotype and lymphoma risk data. Therefore, undetected biases could arise from measurement errors or unaccounted confounders.

Future research should aim to validate these findings through functional studies and clinical trials to elucidate the mechanistic pathways through which these immune cell signatures influence lymphoma risk. Additionally, exploring the temporal dynamics of these associations could offer deeper insights into the progression from immune dysregulation to lymphoma development. The potential for personalized medicine, leveraging these immunophenotypes as biomarkers for risk stratification and tailored immunotherapy, presents an exciting frontier in lymphoma research.

In conclusion, our study adds valuable evidence to the growing understanding of the immune basis of lymphoma. By identifying specific immunophenotypes associated with HL and NML risk, this work paves the way for further investigations into immune-centric therapeutic strategies and underscores the importance of precision medicine in oncology.

## Supplementary Material

Supplementary Table 1

Supplementary Table 2
